# Drivers of innovation value: simulation for new drug pricing evaluation based on system dynamics modelling

**DOI:** 10.3389/fphar.2025.1474856

**Published:** 2025-01-22

**Authors:** Qian Xing, Wendi Cheng, Wei Wang, Chunlin Jin, Haiyin Wang

**Affiliations:** Department of Health Technology Assessment, Shanghai Health Development Research Center (Shanghai Medical Information Center), Shanghai, China

**Keywords:** system dynamics, drug prices, innovation premium, value-based pricing, pricing evaluation

## Abstract

**Objectives:**

Paying for the innovative value of drugs is an important means of mitigating healthcare system duplication and enhancing patient health. Assessing and exploiting the factors influencing innovation premium to forecast trends and shortcomings within the pharmaceutical innovation ecosystem.

**Methods:**

Utilizing system dynamics, this research constructs a decision evaluation system for new drug pricing in Japan. It integrates various decision-making factors across dimensions such as value premium, marketability premium, pediatric premium, and SAKIGAKE premium, employing Vensim PLE software for simulation purposes.

**Results:**

Under the current policy framework, pharmaceutical innovation is on the rise, with significant policy effects observable after 5 years. The most substantial growth in value occurs in medications for rare diseases and niche markets, with effects varying in the short to medium term and stabilizing over the long term. Sensitivity analysis highlights that factors like combination therapies, faster mechanisms of action, and novel therapeutic parts notably influence the value dimension. Other significant factors include obtaining national certifications, addressing indications lacking standard treatments, and demonstrating superior efficacy. The study also identifies underexploited opportunities related to the use of evidence in pricing decisions.

**Conclusion:**

Clinical outcomes are pivotal in shaping drug pricing, influencing both patient and healthcare provider preferences, and thereby affecting market uptake and competitive dynamics. Regulatory frameworks that prioritize unmet medical needs or superior drug efficacy are essential. Future enhancements to the model should incorporate more real-world evidence and expand regulatory considerations to better reflect the dynamic nature of the healthcare sector and support equitable, outcome-based drug pricing.

## 1 Introduction

Over the past few decades, the global burden of disease has escalated alongside healthcare costs, driven by demographic shifts, increasing patient expectations, and the introduction of costly new drugs and healthcare technologies aimed at addressing unmet medical needs (Dimasi et al., 2016). However, the development of new drugs is a key factor in advancing public health (Vokinger et al., 2022). In 2023, global pharmaceutical expenditures were approximately $1.5 trillion, with global spending on medicine using list prices having grown by 35% from 2018 to 2023, and it is forecast to increase by 38% through 2028 (Iqvia, 2024). Given these forecasts, the financial sustainability of health systems is increasingly a concern, as most countries primarily fund these systems through public sources. To manage these challenges, governments worldwide have implemented policies aimed at enhancing the accessibility and affordability of medications, focusing on value-based payment models for innovations (Jommi et al., 2020a; Chalkidou et al., 2020; Jommi et al., 2020b).

The pricing of innovative drugs is an extremely complex issue, which requires both ensuring that firms receive a reasonable return on their R&D investments and guaranteeing fair access to drugs (Incze et al., 2022). This process should not only follow market-driven principles, but also be guided by appropriate policies to regulate market behaviour (Smith, 2022). Governments, health technology assessment (HTA) agencies, and healthcare decision-makers use pricing incentives throughout the entire lifecycle of a drug to encourage the development of truly new medications (Daalen et al., 2021; Hwang et al., 2022). The criteria used for pricing and reimbursement include humanistic, clinical, and economic aspects (Kaltenboeck and Bach, 2018; Levaggi, 2014). To recognize significant innovations in pharmaceuticals, decision-making bodies must explicitly or implicitly define what characteristics constitute “rewardable” innovation (Levaggi and Levaggi, 2024a). Ideally, if countries could agree on this definition, it would provide consistent incentives for manufacturers to research new methods for treating diseases, and simplify drug development, thereby reducing costs and prices (Annemans, 2023). Reasonable value-based pricing decisions have a “spillover effect” on the future innovation ecosystem (Levaggi and Levaggi, 2024b).

Many scholars have conducted systematic reviews on the definitions of drug innovation, dimensions of innovation, and incentive measures across different countries (de Solà-Morales et al., 2018; Wakutsu et al., 2023), These reviews include certifications for rare diseases, pediatric populations, and other exclusive values, as well as validations of the effectiveness of translating new mechanisms of action into actual clinical benefits. Countries have also incorporated elements of innovation into the pricing decisions for new drugs (Prieto-Pinto et al., 2020; Gonçalves, 2022). Germany categorizes the clinical benefits of new drugs into five levels, and patents with high additional benefits are priced higher than the reference drugs (Theidel and von der Schulenburg, 2016). France uses the SMR (Service Médical Rendu) rating to determine different reimbursement rates based on the level of clinical benefit, and then uses the ASMR (Amélioration du Service Médical Rendu) rating to decide the pricing negotiation methods based on the degree of improvement in clinical benefits (Kergall et al., 2021). However, a consistent framework for innovation-based pricing has not been universally established (Zozaya et al., 2024).

Japan is recognized as an innovation-rewarding market with predictable pricing potentials. Drug prices are influenced by market competition, international benchmarks, and government regulations (Mamiya and Igarashi, 2021). Japan’s current National Health Insurance (NHI) drug pricing system is a fast route to market access, and consequently accessed by patients around 3 months after approval, resulting in one of the quickest market access pathways in the world (Takayama and Narukawa, 2017). Since 2014, Japan’s new drug pricing system involves selecting a comparable existing drug and calculating the reimbursement price of a new drug based on established government rules, with premiums awarded for meeting specific criteria (Mamiya and Igarashi, 2021). Although these pricing processes do not comprehensively reflect the broader values that drugs can provide to patients and society, but to a certain extent, it covers various elements of new drug value determination (Takami et al., 2023).

Recognizing the value of new drugs is pivotal for the development of the pharmaceutical innovation ecosystem (Lamattina, 2022). System dynamics (SD) is an established simulation methodology used to explore the behaviour of social systems over time (Lyons and Duggan, 2015). This paper utilizes the SD modelling to analyze the decision-making process for new drug pricing, using Japan’s rating criteria as a case study. The goal is to quantitatively examine how changes in mechanisms of action, national certifications, and other key factors influence the recognition of innovation value and inform policy-making decisions in the pharmaceutical innovation ecosystem.

## 2 Methods

### 2.1 Applicability analysis of SD model

Health policy evaluation systems are inherently complex, consisting of multiple tiers of interdependent subsystems and processes that are adaptive to changes in the environment and behave in a nonlinear fashion. Traditional health technology assessment and modeling methods often neglect the wider health system impacts that can be critical for achieving desired health system goals and are often of limited usefulness when applied to complex health systems (Marshall et al., 2015).

SD is a science that combines system management science with computer simulation and has feedback structure and dynamic reflection (Randers, 1997). It can simplify the actual operating conditions of the research object and provide operable information to decision-makers concisely. Generally, the application of SD requires the system to have certain characteristics and conditions, such as clear boundary, dynamic law, and predictability. Many studies have explored assumptions, hypotheses, and policy at the conceptual/theoretical level and can be characterized to be mostly exploratory modelling tools (Uriona and Grobbelaar, 2019).

The core elements of SD are feedback, accumulations (stocks), rates (flows), and time delays. Stocks are accumulations or aggregations of something (e.g., people, beds, and oxygen). Flows are rates, these feed in and out of stocks and have the same units of stocks per time unit (e.g., people per hour, beds per year, and oxygen per minute). An important concept in SD is nonlinearity. This concept is tied to the existence of feedback processes. It means that an effect is seldom proportional to the cause (Martinez Moyano and Richardson, 2013).

The principle of System Dynamics (SD) emphasizes the correctness of the model structure rather than the precision of input parameters. The polarity of SD feedback loops is insensitive to minor discrepancies in inputs, and the model structure is relatively stable. As long as the data falls within a certain range, the system will exhibit the same behavioral patterns, showing a high degree of tolerance (Bala et al., 2017). When establishing a system model, the primary focus is on ensuring that the described system structure aligns with the actual situation, without excessively concentrating on the selection and precision of parameters. The parameter inputs in this study are not fixed values, making the model suitable for exploring the developmental trends of the dependent variables under different scenarios (Ghaffarzadegan et al., 2011).

### 2.2 Factors influencing pricing premiums for new drugs

Japan implements a universal health insurance system, and in terms of drug price management, it adopts a method that combines government pricing with the healthcare insurance system, with prices uniformly set by the Ministry of Health, Labour and Welfare. This system includes categorized management, price control, and a post-market re-evaluation mechanism. Different pricing methods are applied to innovative drugs and generic drugs. Innovative drugs here are drugs with New Molecular Entity (NME) (Takayama and Narukawa, 2017). For innovative drugs, prices are primarily determined either by comparison with similar drugs or based on cost accounting. When there is a comparable drug with same indication in the list, the daily price of a new drug is determined so that it is same as the daily cost of the comparable drug. Premiums below are applied when the new drug is proven to be highly useful.

**FIGURE 1:**
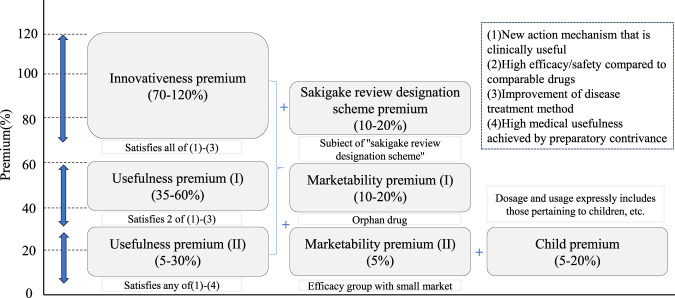
Corrective premiums for new pharmaceuticals in Japan.

Figure 1 shows all premiums, minimum 5% and maximum 120% value premium (breakthrough mark-up), plus possible marketability premium (5%–20%), possible SAKIGAKE review premium (10%–20%), possible paediatric premium (5%–20%). SAKIGAKE Designation System is used to promoting R&D in Japan aiming at early practical application for innovative pharmaceutical products, medical devices, and regenerative drugs.

Under the main VALUE Premium dimension, the core pricing decisions are four issues: (1) New action mechanism that is clinically useful. (2) High efficacy/safety compared to comparable drugs. (3) Improvement of disease treatment method. (4) High medical usefulness achieved by preparatory contrivance. Japanese social security has its own criteria for recognition, Table 1 reflects the boundaries and variables of the four issues.

**TABLE 1:** Main Issues and its variables.

Main issues	Variables
New clinically useful mechanism	New action part, new target, serious diseases without standard treatment, additional clinical significance of NHS accreditation
More effective/safe than comparable drugs	Validity better than comparator, safety better than comparator, evidence from RCT, evidence from other, additional clinical significance of NHS accreditation
May improve disease or injury	Favouring patient proups with poor outcomes, becoming a standard treatment option, faster onset/longing effects, combination to enhance effectiveness, no standard in disease areas, additional clinical significance of NHS accreditation
Formulation improvements to enhance utility	Reducing invasiveness of drug delivery, easy administration, stable blood level, additional clinical significance of NHS accreditation

**FIGURE 2:**
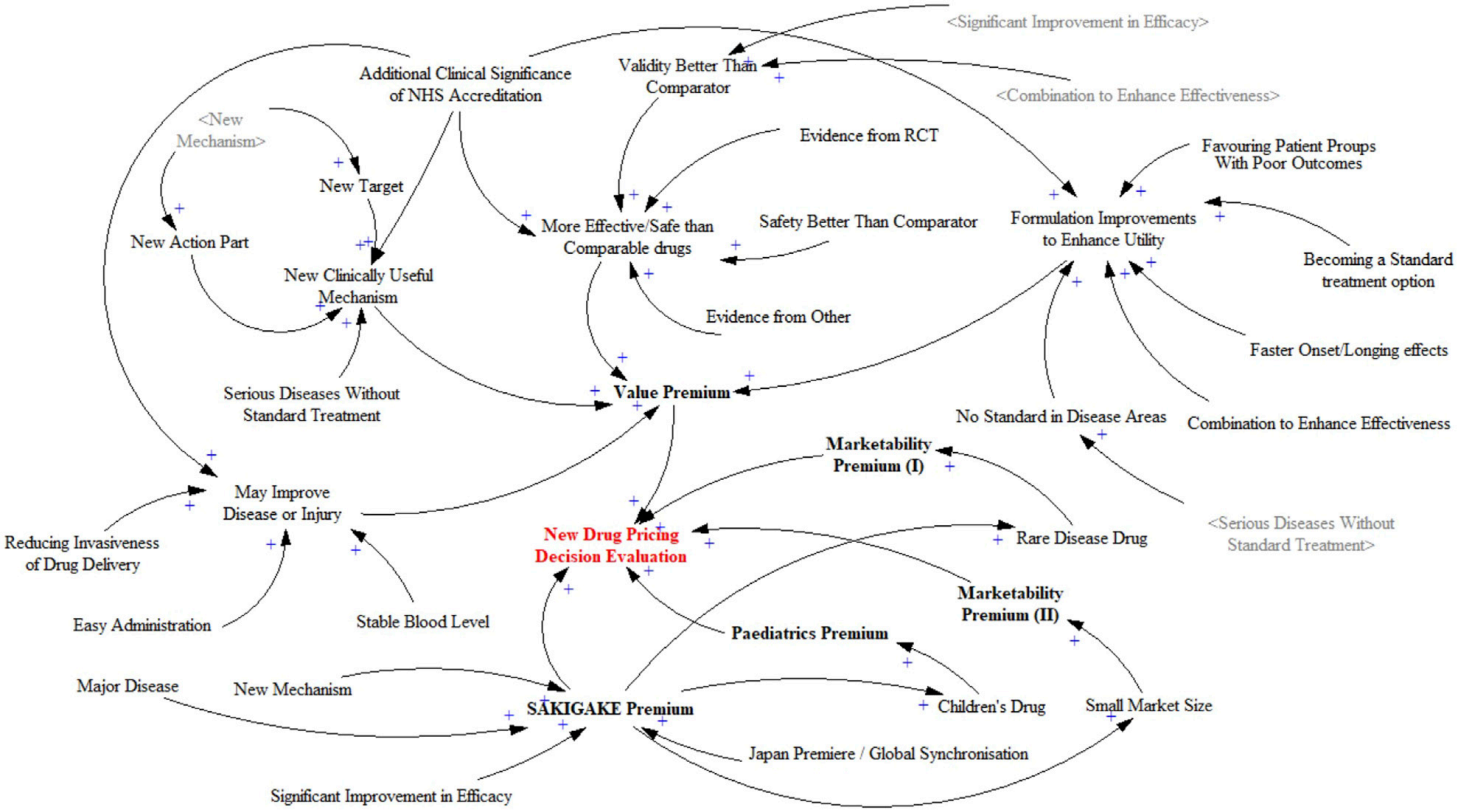
Causality diagram of new drug pricing decision evaluation system.

The primary objective of causal relationship analysis is to delineate the system levels and structures, and to identify the main feedback mechanisms between the overall system and its components. The additive rules for new drug pricing decisions are divided into five subsystems, and the factors influencing each are organized. Each variable’s score contributes to a higher pricing decision. The causal relationship diagram constructed is shown in Figure 2. The New Drug Pricing Decision Evaluation aggregates the impacts of the Value Premium, Marketability Premiums I and II, Paediatrics Premium, and SAKIGAKE Premium. The factors surrounding the five subsystems are each scoring item. One factor has an impact on the others. Variables are generally independent factors in decision-making, but there are interactions and relationships between them. For example, an early certification of a “New Mechanism” directly affects the “New Action Part” and “New Target” during the pricing process, thus creating a positive feedback loop. This interplay exemplifies how variables not only act independently but also influence each other, dynamically affecting the pricing mechanism and ultimately the valuation of the drug’s innovative qualities. The overall relationship diagram reflects a positive relationship, as higher premiums indicate greater value, innovation, and marketability, justifying higher pricing.

### 2.3 Construct SD flow and assign scores

**FIGURE 3:**
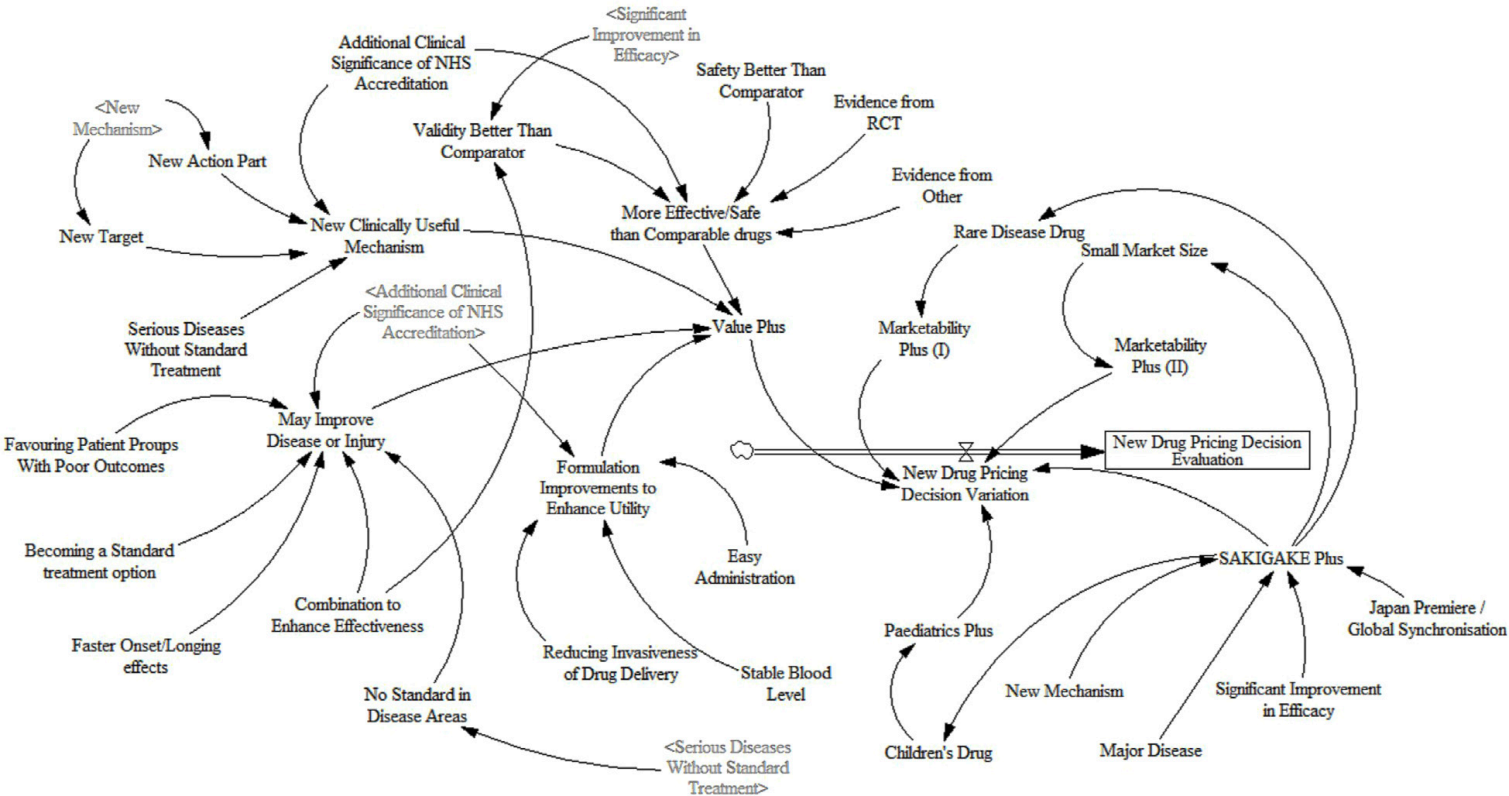
Dynamic flow chart of new drug pricing decision evaluation system.

Based on the causality diagram and the feedback between various factors, this paper introduces 1 stock variables, 33 auxiliary variables, and 17 constant variables to construct the dynamic flow chart of the new drug pricing decision evaluation, as shown in Figure 3.

For SD equation design and variable assignment, scoring rules, comprehensive estimation, and experimental adjustment are adopted. Since there is no fixed unit for scoring, this paper makes it dimensionless (Shi et al., 2022). The simulation step size for the model is set to 1 year, with a total simulation duration of 50 years. Considering the specific characteristics of new drug pricing decisions, reasonable settings for nonlinear functions are applied to the relevant variables within the system. Some variables exhibit certain time lags, which are appropriately configured using information delay functions. The design of equations in this study draws upon a literature review of healthcare policy implementation (Lyons and Duggan, 2015). The model equations are shown in Table 2.

**TABLE 2:** Design of the SD equation.

Variable	Equation design
New action part	2 + new mechanism
New target	1 + new mechanism
Serious diseases without standard treatment	1
Additional clinical significance of NHS accreditation	1
Validity better than comparator	2 + DELAY1(significant improvement in efficacy, 0.5) + combination to enhance effectiveness
Safety better than comparator	1
Evidence from RCT	2
Evidence from other	1
Favouring patient proups with poor outcomes	1
Becoming a standard treatment option	1
Faster onset/longing effects	1
Combination to enhance effectiveness	1
No standard in disease areas	1 + Serious diseases without standard tTreatment
Reducing invasiveness of drug delivery	1
Easy administration	1
Stable blood level	1
Rare disease drug	5 + DELAY1(SAKIGAKE Plus, 5)
Small market size	5 + DELAY1(SAKIGAKE Plus, 5)
Children’s drug	5 + DELAY1(SAKIGAKE Plus, 5)
New mechanism	2
Major disease	2
Significant improvement in efficacy	2
Japan premiere/global synchronisation	2
New clinically useful mechanism	Additional clinical significance of NHS accreditation + new action part + new target + serious diseases without standard treatment
More effective/safe than comparable drugs	Additional clinical significance of NHS accreditation + safety better than comparator + validity better than comparator + evidence from RCT + evidence from other
May improve disease or injury	Additional clinical significance of NHS accreditation + Becoming a standard treatment option + favouring patient proups with poor outcomes + no standard in disease Areas + combination to enhance effectiveness+ “faster onset/longing effects”
Formulation improvements to enhance utility	Reducing invasiveness of drug delivery + easy administration + stable blood level + additional clinical significance of NHS accreditation
Value premium	Formulation improvements to enhance utility + May improve Disease or injury + new clinically useful mechanism+ “More effective/safe than comparable drugs”
Marketability premium (I)	Rare disease drug
Marketability premium (II)	Small market size
Pediatrics premium	Children’s drug
SAKIGAKE premium	New mechanism + significant improvement in efficacy + “japan premiere/global synchronisation” + major disease
New drug pricing decision variation	1.8*Value plus + 1.15*“marketability plus (I)” + 1.05*“marketability plus (II)” + 1.125*paediatrics plus + 1.15*SAKIGAKE plus
New drug pricing decision evaluation	INTEG (new drug pricing decision variation, 0)

## 3 Results

### 3.1 Model validity test

Model validity means that the model can accurately rep resent the actual system, and all simulation models need to be tested for validity. There are many ways to test the SD model, but due to the complexity of the research problem, the model cannot be connected with the real data. Therefore, the verification of the model focuses on whether the model is consistent with the actual trend, in other words, whether the model can produce “reason able” results.

#### 3.1.1 System boundary inspection

One of the keys to the feasibility of the SD model is whether the model has a clear system boundary. The variables and time span in the model will affect the system boundary. Therefore, it is necessary to test the system boundary of the important conceptual variables in the model. The object of this paper is a system for evaluating new drug pricing decisions. The variables used in the model are from publicly available, repeatedly discussed and validated influences. All variables are core variables. Therefore, the SD model established in this paper is effective.

#### 3.1.2 Stability check

**FIGURE 4:**
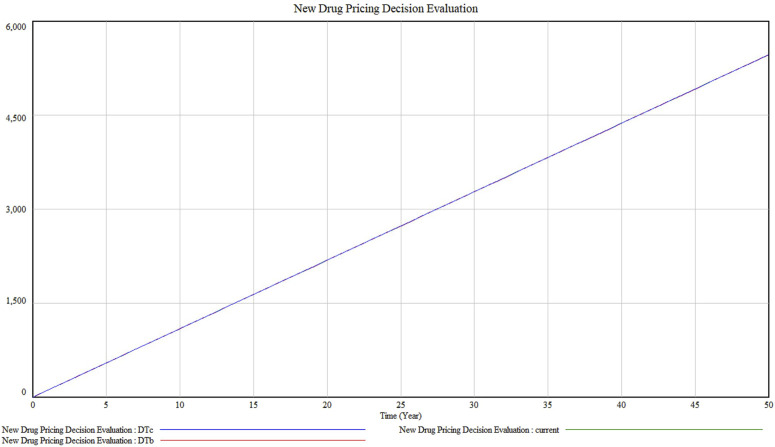
Model stability test result.

Taking the pricing decision system under the existing technical conditions, resource affordability and policy environment, the model stability test can be realised by the integral error test. By setting different simulation time intervals, it is tested whether the operation results of the model are sensitive to the choice of the difference step size. Adopting the way of halving the simulation time interval one by one, i.e., setting DTa = 1 (current), DTb = 0.5, DTc = 0.125 three simulation steps to simulate the model respectively, the results are shown in Figure 4. It can be seen that the pricing decision system is almost unchanged, indicating that the integration error of the model in this paper is small and negligible. After changing the simulation step of the model simulation, the simulation curve has no change, which represents the model stability is good.

### 3.2 Decision evaluation under the baseline scenario

**FIGURE 5:**
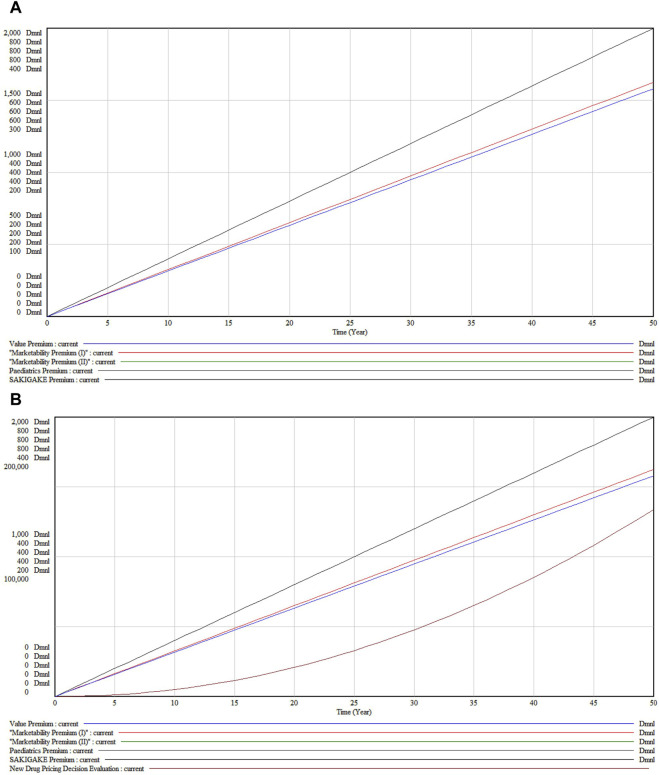
**(A)** New Drug Pricing Decision Evaluation under the baseline scenario **(B)** Accumulation state under the baseline scenario.

Under the baseline scenario as shown in Figure 5A, without changes in external conditions, all premiums demonstrate a linear growth in value over 50 years, indicating expected consistent growth under the pay-for-value system.

Viewed in different subsystems, the Value Premium (Black Line) exhibits the highest growth, reflecting significant long-term appreciation in drug effectiveness and utility. The Marketability Premiums (I and II) (Red Lines), although showing substantial growth, increase at a less steep rate than the Value Premium. The Pediatric Premium (Green Line) displays the least steep growth curve, suggesting a more moderate increase in value for pediatric applications. The SAKIGAKE Premium (Blue Line) shows moderate growth, indicative of its mid-level influence on pricing, likely due to benefits from regulatory acceleration or fast-track approvals.

Figure 5B, depicting cumulative values, contrasts with Figure 5A which illustrates the immediate outputs and dynamics of system components. The cumulative graph demonstrates that all premiums steadily accrue value, influenced by factors such as inflation, market demand, regulatory changes, or medical advancements. The proximity of the trajectories for Marketability Premiums (I and II) implies comparable long-term returns, despite slight variations in short-to medium-term paths. Notably, the Pediatric Premium, initially below the SAKIGAKE Premium, tends to converge and may exceed it in later years, reflecting potentially shifting market dynamics and an increasing emphasis on pediatric health and drug development incentives.

### 3.3 Single factor sensitivity analysis

Using the established model, the sensitivity of various parameters was tested by adjusting the values of specific variables by 80% to observe system responses. This one-factor sensitivity analysis aimed to identify key variables influencing the system, with each simulation altering only one parameter at a time. The results indicate that increasing the value of each constant parameter generally leads to an upward trend in the system’s output, demonstrating a degree of sensitivity to these changes. However, over a 50-year span, most output lines remained closely aligned, suggesting that the decision model is relatively stable or insensitive to changes in a broad range of factors. This stability is crucial for ensuring model robustness against input variations.

Notably, after about 30 years, slight divergences appear among some of the output lines, hinting that certain factors may exert a more significant impact over the long term, or that aspects of the drug’s market dynamics or health impacts evolve with time.

**FIGURE 6:**
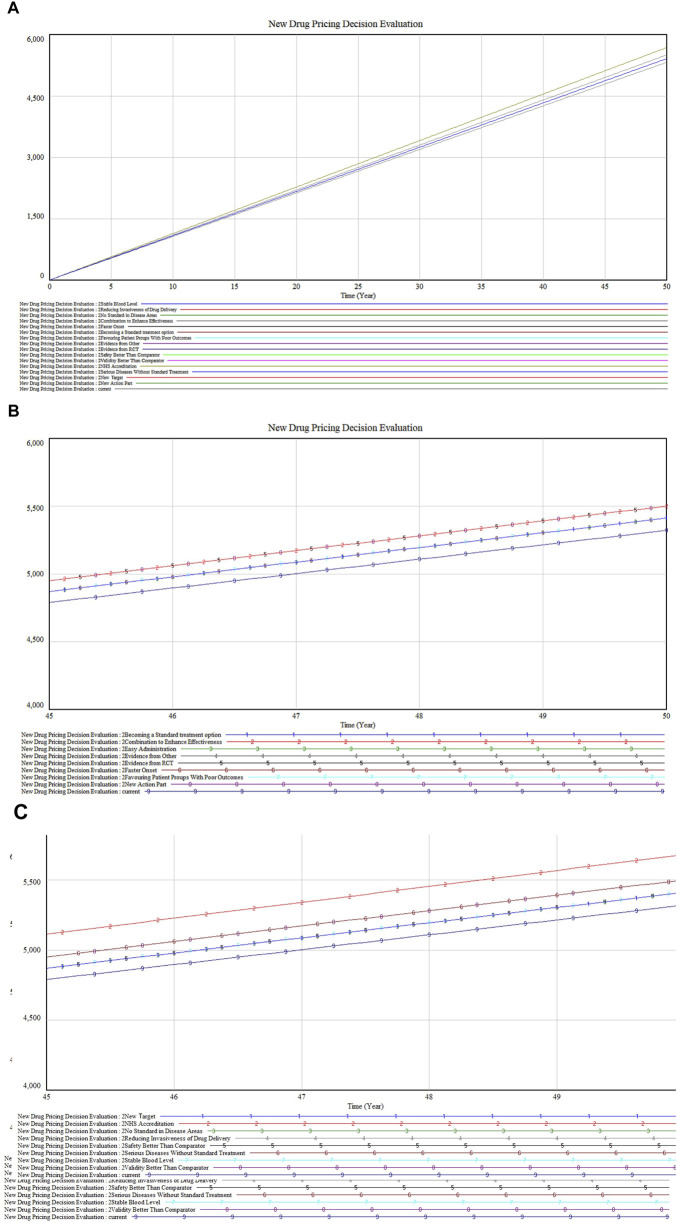
**(A)** Value premium inputs sensitivity analysis **(B)** Partial inputs sensitivity analysis **(C)** Residual inputs sensitivity analysis.

The model’s sensitivity to constants was examined in three segments, with the value premium subsystem involving adjustments to 16 constants. Figure 6A displays the overall impact of these variables and includes a detailed zoom-in plot. In Figure 6B, variables such as “Combination to Enhance Effectiveness” (Marked 2), “Faster Onset” (Marked 5), and “New Action Part” (Marked 8) show overlapping sensitivities and a more pronounced upward trajectory, suggesting that drugs with enhanced combination effects, quicker action, or new mechanisms notably distinguish themselves in the market. Clinically, such treatments are preferred for their rapid relief and innovative approaches.

Figure 6C reveals that “NHS Accreditation” (Marked 2) shows the highest sensitivity, followed by “Serious Diseases Without Standard Treatment” (Marked 6) and “Validity Better Than Comparator” (Marked 8). These factors are crucial for gaining market access and favorable reimbursement conditions. Drugs addressing unmet medical needs or demonstrating superior efficacy not only deliver significant clinical benefits but also achieve economic advantages through market exclusivity and reduced competition.

**FIGURE 7:**
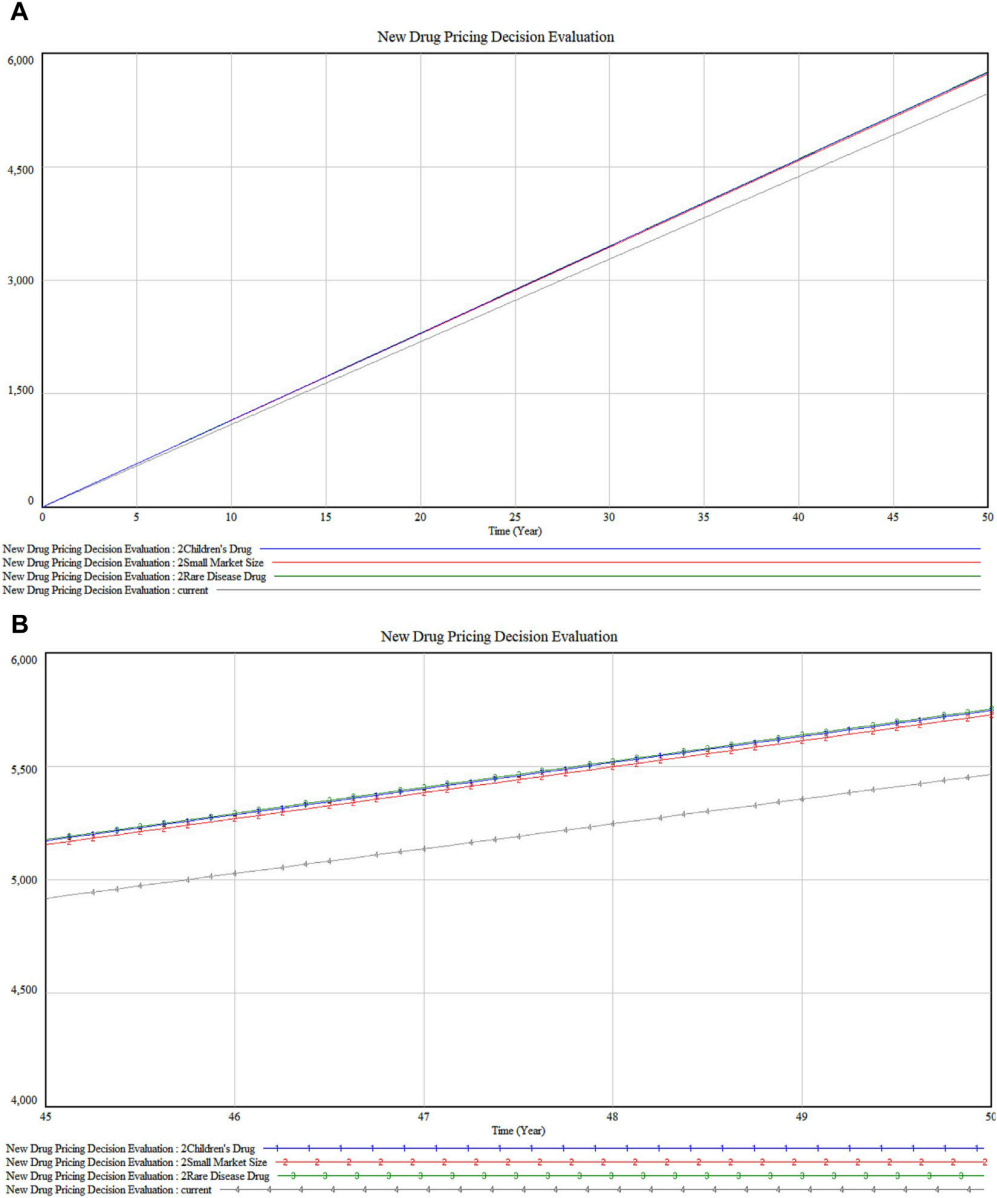
**(A)** Special Dimension Premium sensitivity analysis **(B)** Detailed version in Special Dimension Premium sensitivity analysis.

In the analysis of premium dimensions as illustrated in Figures 7A, B, drugs targeting rare diseases (marked 3) exhibit a slightly higher impact on marketability compared to children’s drugs (marked 1). This differential could stem from the unique challenges in rare diseases such as the absence of alternative treatments and the substantial enhancement in patient quality of life these drugs provide. Furthermore, the close impact level of children’s drugs reflects the societal and ethical emphasis on pediatric healthcare. Both categories show parallel trends with minor fluctuations but maintain proximity in their impact values. Following these are drugs with “small market size” (marked 2), which may address conditions of lesser severity or urgency, or attract less public and medical attention compared to rare disease or children’s drugs.

**FIGURE 8:**
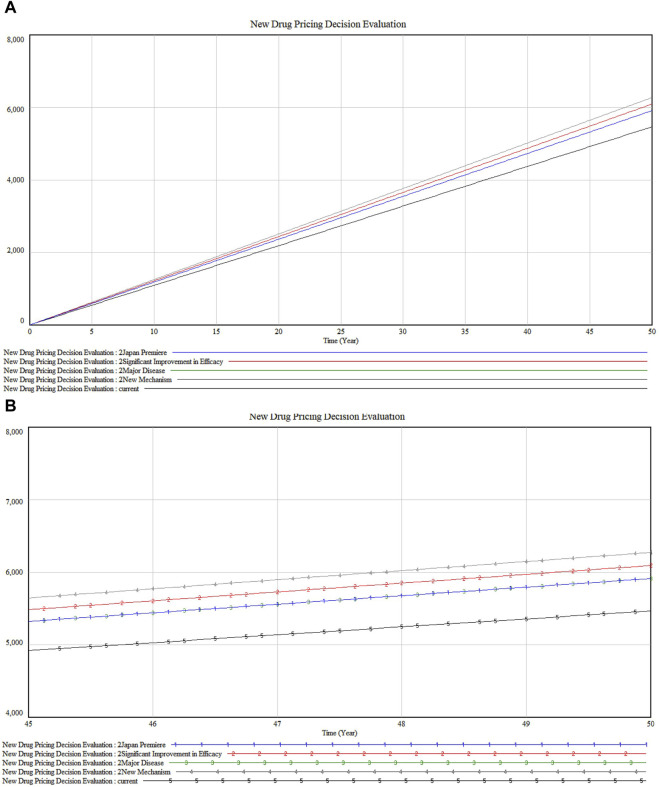
**(A)** SAKIGAKE Premium sensitivity analysis **(B)** Detailed version in SAKIGAKE Premium sensitivity analysis.

Further exploration into the SAKIGAKE Premium certification reveals the dynamics among four constants in Figures 8A, B. “New Mechanism” (marked 4) leads in impact, indicating its role in pioneering new treatment avenues. This is closely followed by “Significant Improvement in Efficacy” (marked 2). The constants “Japan Premiere” (marked 1) and “Major Disease” (marked 3) display nearly identical impacts, underscoring the value placed on first-to-market and major disease-targeting drugs in Japan. These findings highlight the premium’s recognition of innovations that significantly advance patient outcomes, correlating with a higher valuation in the market.

## 4 Discussion

This paper constructs the SD model for evaluating new drug pricing decisions, integrating five subsystems: value premium, marketability premiums I and II, paediatrics premium, and SAKIGAKE premium. The model’s validity tests confirm its ability to accurately reflect the impact of key factors, providing a robust foundation for simulation results. Under a baseline scenario, all premiums linearly increase over 50 years, suggesting a continuous rise in drug values within a pay-for-value system. This reflects the model’s assumption that drug innovations will remain valuable, encouraging sustained investment in pharmaceutical innovation.

The distinct growth trajectories of the premiums, particularly the pronounced increase in the Value Premium, highlight the critical role of drug efficacy and utility in pricing. This aligns with the shift towards value-based pricing models in healthcare, which prioritize clinical outcomes. Sensitivity analysis reveals that innovations enhancing drug synergy, speed of action, and introducing novel mechanisms are highly valued, guiding pharmaceutical companies to focus their R&D on these areas.

Further, the high sensitivity associated with “NHS Accreditation,” “Sensuous Diseases Without Standard Treatment,” and “Validity Better Than Comparator” reinforces the importance of regulatory endorsement and addressing unmet medical needs. This supports the idea that drugs fulfilling these criteria can achieve premium pricing and suggests that regulatory strategies and drug development targeting these areas can be particularly effective. The analysis of marketability and special premiums, like those for rare diseases and pediatric applications, provides nuanced insights into market dynamics. The higher impact of “rare disease drug” over “children’s drug” might reflect the urgent need and lack of alternatives in rare diseases, which often allows for premium pricing under orphan drug status (Zelei et al., 2021).

From a regulatory and health economics perspective, these sensitive factors often resonate well with regulatory agencies and health economics assessments. Drugs that show superior effectiveness, rapid onset, or novel actions might receive favorable formulary placements and coverage decisions, which can influence pricing positively. From a clinical perspective, treatments that offer rapid relief, enhance the effects of existing therapies, or provide new treatment avenues are likely to be preferred by both patients and providers. This preference can translate into higher willingness to pay, which can be leveraged in pricing strategies (Olsder et al., 2023). These factors of pricing decisions guide the R&D of pharmaceutical industry. Pharmaceutical companies might be pricing these drugs not solely based on market demand but also considering the necessity and the potential for significant patient benefit. This could be part of a broader strategy to align with regulatory incentives and societal expectations, particularly in areas like rare diseases where patient advocacy and public interest are strong.

In this pricing decision evaluation model, we can also find that the sources of evidence that have received more attention in recent years have not been sufficiently influential, one because of their own small scores and the other because of their low correlation with other factors. Medicare payments for post-marketing drugs can be enabled by generation of real-world evidence (RWE) utilising robust real-world data (RWD) to enable drug payments based on actual patient value received rather than what the healthcare system value hoped for (Bn et al., 2015; Eichler et al., 2022). If its focus is to be strengthened at the time of pricing, it could be explored to delineate the level of evidence for RCTs, highlighting links to efficacy, and whether the RCT produces the desired outcome, *etc.* (Chan et al., 2020). In fact, the new HTA methodology, recently published in the United Kingdom by NICE (Dawoud et al., 2022), introduces a weighting factor for serious diseases so that the absolute value of the difference between the QALYs of the two groups or the percentage difference between the QALYs can be used to quantify the severity of the disease when comparing clinical trials and the principle of opportunity cost neutrality is applied to redistribute different weights to different diseases. The “opportunity cost neutrality” principle is used to reallocate different weights to different diseases (Angelis et al., 2023). Also included are uncertainty about the likelihood of treatment, scarcity, equity, age, and information that allows for clinical evidence of innovative technologies, which could be considered for integration into the pricing of new drugs to create a linkage.

In addition, the current analyses of price premium factors for new drug launches have not yet taken into account the issue of price adjustments due to changes in the competitive environment after launch, and future studies of price mechanisms involving the whole life cycle of drugs will face more adjustments (Fu et al., 2018). In terms of related institutional research, policy externality issues such as the spillover effects of price policy and other responsibilities that price policy itself needs to assume will make the analysis more complex.

In 2018, the International Society for Pharmacoeconomics and Outcomes (ISPOR) Special Task Force considered the elements of value in healthcare and identified a series of elements, the so-called ISPOR value flower. Although the ISPOR value flower advocates including a broader range of value elements, there are challenges to its universal use because several elements included in the ISPOR value flower such as value of insurance, severity of disease, value of hope, and value of real option are not clearly defined. All these elements are considered to be important, but a method for quantifying them in monetary terms has not yet been established (Lakdawalla et al., 2018).

System dynamics belongs to the classic cross-comprehensive discipline, which on the one hand can systematically analyse the interactions among various premium factors and clearly present the degree of elemental correlation, and on the other hand, it can also simulate and evaluate the trend of the factors from the dynamic perspective, making the results more interpretable. At present, the SD model has limited application in the field of health (Atkinson et al., 2015), after which it can be considered to expand its application in the policy decision-making, as an effective tool for clarifying the relationship between various types of variables and clarifying the degree of criticality.

This study provides actionable insights for policymakers and regulatory agencies by presenting a comprehensive evaluation of factors influencing new drug pricing. It clarifies the trends and shortcomings of Japan’s current drug pricing framework compared with the goal of value-based healthcare, providing a reference for Japan and other countries. Of course, there are limitations to this study as an exploratory study. While the model demonstrates stability and sensitivity to critical variables, the lack of internationally recognised scoring values limit the accuracy of the simulations. Future research could aim to integrate synthesised scoring data as it becomes available, possibly adjusting the model to reflect real-world complexities more accurately. Moreover, exploring additional scenarios where external factors such as economic downturns or changes in healthcare policy significantly alter drug pricing dynamics could provide deeper insights into the resilience and adaptability of the pricing strategies under different market conditions. Finally, the SD model is inherently limited in that the model does not require a high degree of parameter accuracy and the interpretation of the results is subjective and needs to be further analysed in the context of real-world situations.

## 5 Conclusion

This study develops a system dynamics (SD) model to evaluate new drug pricing decisions by integrating key premium factors such as value, marketability, paediatrics, and SAKIGAKE premiums. In Japan, the inclusion of a structured pricing system provides a relatively efficient, transparent, and predictable pathway for new drugs. Nevertheless, there are potential areas where changes could improve access, efficiencies, and value, such as weighting of disease specificity, level of evidence source, and other broader pricing factors in the longer term. These modifications will help guide health systems to balance innovative incentives and health system sustainability to support better and more equitable use of healthcare resources.

## Data Availability

The original contributions presented in the study are included in the article/supplementary material, further inquiries can be directed to the corresponding author.
